# Agnus-Castus

**Published:** 1896-05

**Authors:** C. L. Olds


### AGNUS CASTUS.

C. L. Olds, M. D., H. M.

The origin of the name of this plant is rather interesting. *Agnus* means a lamb, and *castus*, chaste. It was called agnus because the leaves have a woolly substance upon them; and castus, because at the celebration of the feast of Ceres the good matrons would place this woolly substance on their beds, that they might be chaste wives.

Farrington says that this remedy is especially applicable to old men, who have spent their lives in sexual excesses, and at sixty or seventy have just as great sexual desire as when eighteen or twenty—but the desire is all mental—physically they are impotent. The provings justify this assertion. The patient is a melancholy, hypochondriacal subject; peevish, absent-minded, incapable of attending to any business, unable to fix the attention upon mental work. He sometimes feels as if he were nobody and would like to be dead, and at other times is exalted, feeling as if he were a great man. He has vertigo—feels as if he were turning in a circle; also tearing headache, above the eyes, with photophobia.

In these old sinners the sexual organs will be cold, flaccid, relaxed, cold to the touch. Voluptuous fancies excite no erection ; although they have the most intense sexual desire mentally, they are unable to have an erection. They have emissions of semen while at stool, or pass prostatic fluid while at stool.

This remedy is sometimes indicated in gonorrhœa, gleet, with thick, yellow discharge, but no erections. This is peculiar, because generally with gonorrhœa there are painful erections, more or less priapism. There may be premature old age as a result of sexual abuses. The testes feel cold to the touch, nothing induces an erection ; there is great weakness, both of body and mind.

A certain man, to prevent producing children, took this remedy in large doses for three months. Afterward he had a great desire for children, but no erections; the semen was lost while at stool or dribbled away.

Agnus castus may be indicated during lactation. The milk is suppressed or scanty. The patient becomes gloomy, sad, melancholy, keeps saying that she will die; she is troubled with sleeplessness, or if she sleeps wakes with a start and gloomy forebodings.

There are troubles in connection with the female genitals; metrorrhagia, lasting from ten to eighteen days; suppressed menses, with parts relaxed ; leucorrhœa in place of the menses; leucorrhœa flows away without her knowing it. She feels as if all the pelvic viscera were sinking, dragging down—wants to support them with the hands. She has constipation.

A peculiar symptom is that the flatus smells like old urine.

There are pains in different joints—in the shoulders, arms, hands, knees—with rheumatic swellings. The remedy has been successfully used in sprains.

There is a sensation at the root of the nose as if it were pressed upon, ameliorated by pressure; also a smell of musk before the nose. There are corrosive itchings in different parts.

This is a thirstless remedy—no thirst in any of the complaints.

The complaints of this remedy are chiefly in the sexual sphere, due to early excesses. This seems to be its principal application.

